# Curcumin Modulates *NOX* Gene Expression and ROS Production via P-Smad3C in TGF-β-Activated Hepatic Stellate Cells

**DOI:** 10.61186/ibj.4005

**Published:** 2023-10-28

**Authors:** Shahla Asadizade, Mahdi Hatami, Samaneh Salehipour Bavarsad, Benyamin Kabizade, Elham Shakerian, Mojtaba Rashidi

**Affiliations:** Cellular and Molecular Research Center, Medical Basic Sciences Research Institute, Ahvaz Jundishapur University of Medical Sciences, Ahvaz, Iran

**Keywords:** Curcumin, Liver cirrhosis, NADPH oxidases, Reactive oxygen species

## Abstract

**Background:**

Liver fibrosis, associated with HSCs, occurs when a healthy liver sustains damage, thereby impairing its function. NOXs, specifically isoforms 1, 2, and 4, play a role in ROS production during hepatic injuries, resulting in fibrosis. Curcumin has shown strong potential in mitigating liver fibrosis. Our research aimed to investigate the effects of curcumin on lowering NOX and ROS levels. This compound was also studied for its effects on NOXs, ROS concentrations through the inhibition of Smad3 phosphorylation in TGF-β-activated human HSCs.

**Methods:**

MTT assay investigated the cytotoxic effects of curcumin on HSCs. The cells were activated by exposure to TGF-β (2 ng/mL) for 24 hours. After activating, the cells were treated with curcumin at 25-150 μM concentrations. After administering curcumin to the cells, we employed RT-PCR and Western blot techniques to evaluate the related gene and protein expression levels. This evaluation was primarily focused on the mRNA expression levels of *NOX1*,* NOX2*,* NOX4* and phosphorylated Smad3C.

**Results:**

The mRNA expression level of aforesaid *NOXs* as well as *α-SMA, collagen1-α*, and ROS levels were significantly reduced following 100 μM curcumin treatment. Furthermore, curcumin significantly decreased the p-Smad3C protein level in TGF-β-activated cells, with fold changes of 3 and 2 observed at 75 and 100 μM, respectively.

**Conclusions:**

Curcumin decreased the levels of ROS and *NOX*, as well as the expression of *α-SMA and collagen1-α*. The primary mechanism for this reduction could be linked to the level of p-Smad3C. Hence, curcumin could serve as an effective therapeutic agent for liver fibrosis.

## INTRODUCTION

Liver fibrosis stems from persistent liver ailments and represents a critical stage in disease progression. This condition primarily arises due to the overproduction and accumulation of ECM. In situations where the synthesis of ECM significantly exceeds its degradation, if this condition persists, liver fibrosis can progress to more severe conditions such as cirrhosis or hepatocellular carcinoma^[^^1^^]^. Until now, factors that trigger liver fibrosis in order to decrease the risk of cirrhosis development and hepatocarcinoma have not fully been understood. Therefore, investigation of these factors seems to be essential ^[^^2^^]^. Recently, attention has been focused on the effect of HSCs on the progression of liver fibrosis^[^^3^^]^. When liver tissue is damaged, HSCs undergo changes in their characteristics, become more proliferative, motile, and contractile, and produce ECM, which all contribute to fibrosis^[^^4^^,^^5^^]^.

Regardless of their origin, recurring injuries cause damage, which leads to the death of parenchymal cells like hepatocytes. The death of hepatocytes results in the release of intracellular contents such as DNA and ROS, which then activates the cells present in the liver, known as star-shaped cells or HSCs. In this state, cells mimic myofibroblasts, leading to an increase in the production of ECM proteins, such as collagen. This production by HSC-derived myofibroblasts causes the accumulation of matrix^[^^6^^]^. At this stage, macrophages and myofibroblasts differentiate into neutrophils and produce additional inflammatory mediators, including tumor necrosis factor alpha, IL-1, and IL-6, as well as profibrogenic substances such as PDGF and TGF-β^[^^7^^,^^8^^]^.

Most HSCs differentiate into myofibroblasts through the secretion of TGF-β. These myofibroblasts also proliferate in response to PDGF. The buildup of ECM, as well as the production of α-SMA and collagen 1, are both enhanced by myofibroblasts that originate from HSCs. Tissue inhibitor of metalloproteinases, a substance produced by myofibroblasts and macrophages, inhibits the protease activity of MMPs. These MMPs, secreted by macrophages, are responsible for breaking down the matrix formed post-healing. As a result of this process, the matrix accumulates and deposits within liver cells, leading to scar formation. In fact, fibrosis is the outcome of a dysfunctional equilibrium between the degradation and buildup of the ECM ^[^^9^^]^.

Hepatic damage and hepatic fibrosis development are mostly caused by oxidative stress. Following an imbalance between pro-oxidant and antioxidant cellular components, ROS are generated, giving rise to the aforementioned damage. The normal metabolism of cells is responsible for generating ROS, a family of pro-fibrotic mediators that includes hydroxyl radicals, superoxides, and hydrogen peroxide. When ROS levels are low, they serve as a messenger to trigger numerous cellular responses. In contrast, when ROS levels are excessive, they can alter cellular DNA, proteins, and lipids and cause liver cell death and necrosis. In addition, ROS can activate HSCs, Kupffer cells, and other pro-inflammatory cells, which in turn increases the production of inflammatory and fibrogenic proteins^[^^10^^]^.

NOX activation is significantly influenced by the cytokine TGF-β via the Smad3 transcription factor^[^^11^^,^^12^^]^. Smad2 is phosphorylated in response to TGF-β, and p-Smad3 subsequently binds to Smad4, causing the translocation of the Smad complex into the nucleus, where it triggers the production of fibrosis genes such as *NOXs* and ECM proteins^[^^13^^]^. Research suggests that blocking the TGF-β signaling pathway by inhibiting Smad3 phosphorylation can effectively and safely reduce liver fibrosis in animal models. Hence, the suppression of the TGF-β/Smad3 signaling pathway could be a successful approach to avert the stimulation of HSCs and diminish liver fibrosis^[^^14^^]^.

Turmeric (Curcuma longa), and especially its primary bioactive component, curcumin, is broadly utilized as a flavoring agent in a wide variety of Asian dishes. Curcumin has been demonstrated to elicit antioxidant, anti-inflammatory, and tumor-fighting activities^[^^15^^,^^16^^]^. Moreover, its beneficial biological effects^[^^17^^] ^are attributed to the ability of curcumin to reduce excessive ROS generation in mammalian cells. In this research, our hypothesis suggests that curcumin might impede *NOX1, NOX2, and NOX4,* as well as ROS expression in human HSCs by inhibiting the activation of TGF-β/phosphorylated Smad3C signaling. With this in mind, we aimed to assess the potential efficacy of curcumin in addressing liver fibrosis.

## MATERIALS AND METHODS

**Cell culture**

The human HSC was acquired by Dr. Scott L. Friedman from the Icahn School of Medicine at Mount Sinai (NY, USA). The cells were grown in cell culture containers with the addition of 10% fetal bovine serum (Sigma, USA). The incubator offered an environment conducive to the growth of the cells and composed of 95% pure air and 5% CO_2_. Human HSCs were stimulated and activated using TGF-β (2 ng/ml). Subsequently, different concentrations of curcumin (0, 25, 50, 75, 100, and 150 µmol/l) were applied to the cells for a duration of 24 hours.

**Cell viability assessment**

The viability of human HSCs was assessed by employing the colorimetric MTT assay, following established protocols^[^^18^^]^. Approximately, 5 × 10^3^ cells were seeded in each well of a 96-well culture plate and incubated for 24 hours. The cells underwent treatment were rinsed two times with PBS (Sigma Aldrich, USA) and then exposed to MTT (Sigma Aldrich) at a concentration of 5 mg/ml in an incubation chamber for 4 hours. Subsequently, DMSO (Sigma Aldrich) was dissolved in each well. The absorbance of the sample was then measured at 570 nm using an ELISA reader (BioTek, ELx800, USA).

**Intracellular ROS detection**

The intracellular ROS levels in human HSCs were assessed using DCFH-DA. The cells were cultured in a serum-free medium, and 10 µmol/l of DCFH-DA was added. The medium was then incubated at a temperature of 37 °C for a duration of 40 minutes. The emission wavelength of 530 nm was utilized to quantify the fluorescence of DCFH-DA using a FACSCalibur flow cytometer (BD Biosciences, USA).

**Quantitative RT-PCR assay**

The RNeasy Mini kit (Qiagen, Netherlands) was employed to extract the total RNA. After isolating the RNA, we performed reverse transcription to synthesize complementary DNA by using the Yekta Tajhiz kit (Tehran, Iran). The quantitative RT-PCR kit protocol (Amplicon, Denmark) was employed to conduct quantitative RT-PCR assay using SYBR Green. Takara (Takara Bio Inc., Japan) provided us with specific primers for the following genes: *αSMA*, *Collagen1α*, *NOX1*, *NOX2*, *NOX4*, and *GAPDH* (as the internal control). The 2^-ΔΔCt^ method was utilized to determine the comparative levels of gene expression.

**Western blot analysis**

HSCs were lysed using RIPA buffer. The protein concentrations in the extracts were quantified using the BCA kit (Sigma Aldrich). The protein samples were separated using SDS-PAGE electrophoresis, with 50 µl of sample loaded into each well. To prevent any unspecific interactions, non-fat milk was employed to block the binding sites. The mixture was then incubated at 25 °C for one hour. Following the washing process, the membranes were incubated with primary antibodies targeting p-SMAD3 (cell signaling Technology, USA) at 4 °C for 12 hours. After washing procedures, the membranes were subjected to a one-hour incubation period at room temperature, using secondary antibodies. The software Image J was utilized to analyze the intensities of the bands.

**Statistical Analysis**

Data are displayed as the mean ± standard deviation. The ANOVA was utilized to determine differences among the various groups. A *p* value below 0.05 was deemed to have statistical significance. 

## RESULTS

**Curcumin hindered the proliferation of HSCs**

**Fig. 1:**
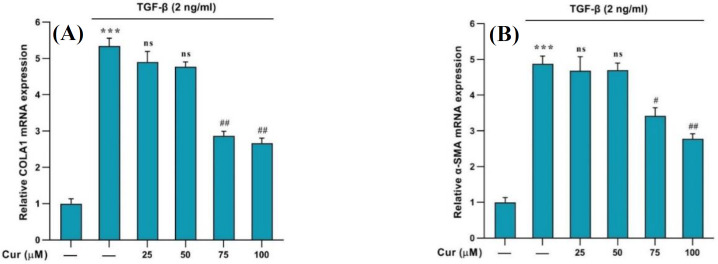
Effects of different concentrations of curcumin on the expression of *COLA1 and αSMA* genes in curcumin-treated cells. Data are shown as the mean ± SEM of three replicates. *GAPDH* was the reference gene. ^***^p < 0.001 vs. vehicle-treated control; ^#^*p* < 0.1 and  ^##^*p* < 0.01 vs. treated control; ns: not significant

**Fig. 2:**
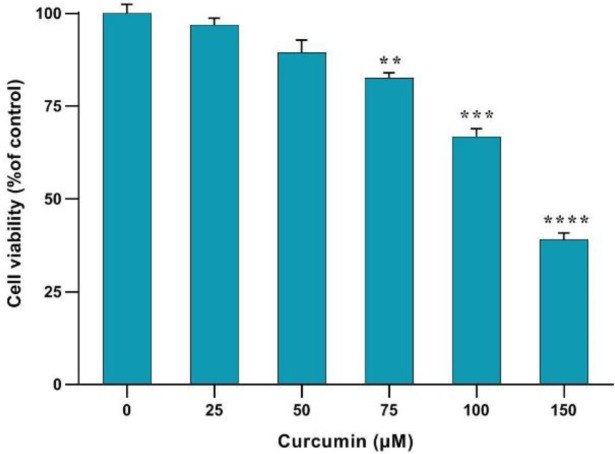
Effects of different concentrations of curcumin on the human HSCs' viability during 24 h. Results are presented as mean ± SEM. ^**^p < 0.01, ^***^p < 0.001, ^****^p < 0.0001 compared to the control

The upregulation of *α-SMA and collagen1-α* genes indicated the activation of HSCs (Fig. 1). Treatment with curcumin (75, 100, and 150 µM) effectively inhibited the proliferation and activation of these human cells. The MTT assay results revealed that curcumin could suppress cell proliferation in a concentration-dependent manner, demonstrating its substantial antiproliferative impact. Concentrations exhibiting cytotoxicity for the cell survival were selected for further experiments by using a concentration less than 150 µM (Fig.2).

**Curcumin diminished the expression of** ***NOXs*** **in TGF-β-activated human HSCs**

**Fig. 3:**
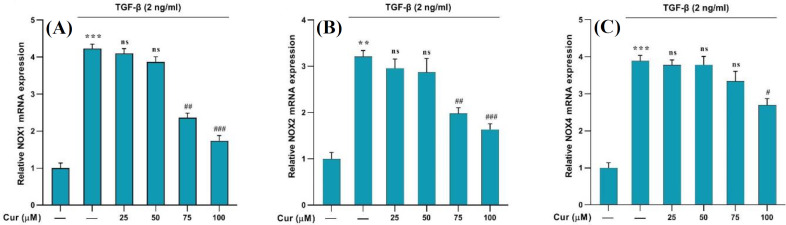
Effects of different concentrations of curcumin on the (A) *NOX1*, (B) *NOX2*, and (C) *NOX4* mRNA gene expression in TGF-β-treated cells. Data are shown as the mean ± SEM of three replicates. *GAPDH* was the reference gene. (^**^p < 0.01 and ^***^p < 0.001 vs. vehicle-treated control; ##*p* < 0.01 and ^###^*p* < 0.001 vs. treated control; ns: not significant

To determine the expression of the *NOX1, NOX2, *and *NOX4* genes, we administered TGF-β (2 ng/ml) to human HSCs for 24 hours. This treatment was accomplished to activate the cells and initiate liver fibrosis, which led to a significant increase in the expression of the aforementioned *NOX* genes. Afterwards, different concentrations of curcumin (25, 50, 75, and 100 µM) were administered to the activated cells. Curcumin exhibited notable efficacy in reducing the expression levels of the three aforementioned genes in the cells treated with 75, and 100 µM of curcumin. Nevertheless, no significant effect was observed at a concentration of 25 µM, indicating a concentration-dependent effect of curcumin (Fig. 3). 

**Curcumin decreased ROS generation in TGF-β-activated human HSCs**

**Fig. 4:**
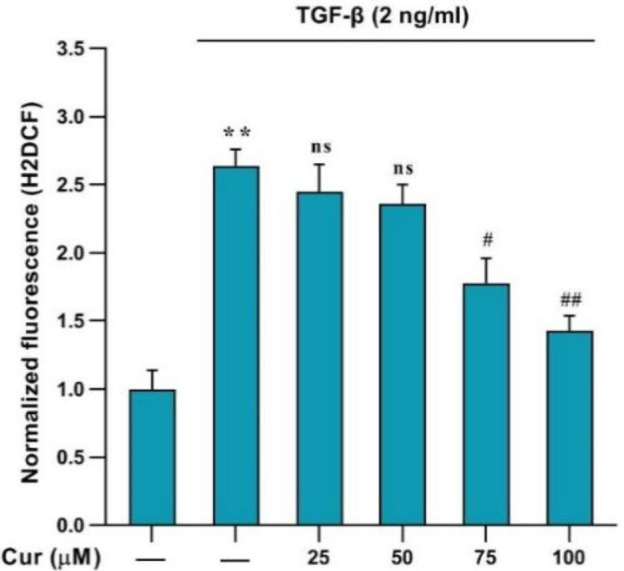
The intracellular ROS levels in human HSCs detected by DCFH-DA. **p < 0.01 vs. vehicle-treated control, #*p* < 0. 1, ##p < 0. 01 vs. treated control. Ns: not significant

Intracellular ROS generation was assessed by DCFH-DA fluorescence in human HSCs using flow cytometry. Post-treatment with curcumin showed a significant reduction in ROS production (Fig. 4). These results indicate the potent antioxidant effect of curcumin, as it appears to reduce the quantity of ROS in cells at the specified concentrations (75 and 100 µM).

**Curcumin lowered p-Smad3C levels in TGF-β-activated human HSCs**

**Fig. 5:**
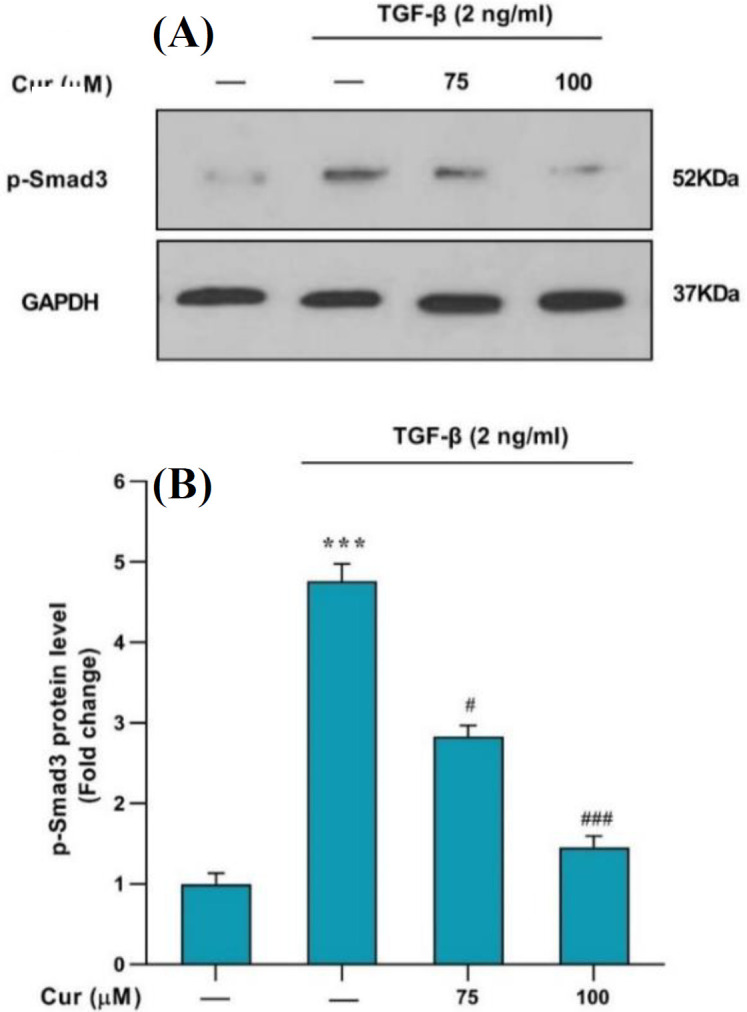
(A) Analysis of the p-Smad2C protein level in TGF-β-activated human HSCs by Western blot. The cells were treated with 75 and 100 μM of curcumin. (B) The relative p-Smad2C level expressed as the ratio p-Smad2C/GAPDH. The data are presented as the mean ± SEM of three replicates; ^***^p < 0.001 vs. vehicle-treated control; ^#^*p* < 0.1 and ^###^*p* < 0.001 vs. treated control

Curcumin was found to inhibit the activation of Smad3C, phosphorylated via the TGF-β pathway, in human HSCs. Western blot analysis was conducted to examine the expression of p-Smad3C in the TGF-β-activated HSCs following the curcumin treatment. Curcumin noticeably inhibited the expression of P-Smad3C in a concentration-dependent manner at 75, and 100 µM. Phosphorylated Smad3C was markedly diminished by curcumin, suggesting that this compound could potentially influence the prevention and activation of human HSCs in liver fibrosis (Fig. 5). 

## DISCUSSION

The increasing global prevalence of liver fibrosis carries the potential to progress to conditions such as cirrhosis and hepatocarcinoma, which could ultimately lead to fatal outcomes if left untreated. This lack of definitive treatment underscores the critical need to identify and investigate potential therapeutic agents^[^^19^^,^^20^^]^. Moreover, the significant absence of effective medications for treating liver fibrosis represents a major challenge in the field of medical science. Therefore, a collective effort by researchers to identify safe and efficacious antifibrotic agents is essential^[^^21^^]^. In light of the urgent need for effective therapeutic agents for liver fibrosis, our study provides promising insights. Our results highlight the potential of curcumin as a viable candidate for this purpose.

Our results showed the upregulation of *α-SMA* and *collagen1-α* genes, indicative of HSC activation, which was effectively inhibited by curcumin treatment. This observation is further corroborated by the MTT results, demonstrating a concentration-dependent suppression of cell proliferation by curcumin and confirming its substantial antiproliferative impact. These findings were aligned with previous research^[^^22^^]^ and indicated that curcumin has a significant inhibitory effect on the activation and proliferation of HSCs. Interestingly, curcumin not only hindered the proliferation of HSCs but also diminished the expression of *NOX *genes in TGF-β-activated HSCs^[^^11^^]^. The significant increase in the expression of *NOX1, NOX2, and NOX4* genes, following TGF-β administration, was notably reduced by curcumin treatment. However, this effect was in a concentration-dependent manner with no significant effect at a concentration of 25 µM. Our results support that curcumin targets multiple pathways to halt HSC activation^[^^23^^]^. Furthermore, our findings displayed that treatment with curcumin leads to a significant reduction in ROS generation in TGF-β-activated HSCs^ [^^5^^]^, as evidenced by DCFH-DA fluorescence assessment using flow cytometry. This outcome suggests that curcumin may exert its antifibrotic effects, at least in part, by mitigating oxidative stress in HSCs. Our findings on the anti-fibrogenic properties in the present study, along with the latest research^[^^24^^]^, confirm the significant antifibrotic effect of curcumin.

Several intracellular signaling pathways are implicated during the development of liver fibrosis; however, the TGF-β-activated pathway plays a crucial role in liver disease development and represents a significant target for therapeutic intervention. During liver injury, TGF-β induces HSC activation, leading to phenotypic changes and increased expression of genes such as *NOX* and ROS levels. Thus, inhibiting HSC activation could be beneficial in the treatment of liver fibrosis^[^^25^^]^. Studies have also provided evidence that flavonoids, due to their antioxidant properties, mitigate liver fibrosis progression^[^^26^^,^^27^^]^.

Overall, the study found that curcumin inhibited the activation of Smad3C, a key player in the TGF-β pathway, in human HSCs. This was shown by a noticeable reduction in the expression of phosphorylated Smad3C following curcumin treatment. This is a novel finding that strengthens our understanding of the mechanisms by which curcumin exerts its anti-fibrotic effects. Our study is consistent with previous findings of Hernández-Aquino et al. who reported that curcumin can downregulate Smad pathways in HSCs in experimental fibrosis^[^^28^^]^.

## CONCLUSION

The present study supports the hypothesis that curcumin impedes the activation of human HSCs by blocking the expression of *NOX* genes and reducing the production of ROS by modulating the TGFβ/SMAD3 signaling cascade. In this study, curcumin significantly reduced the mRNA expression of *NOX1*, *NOX2*, *NOX4*, as well as *α-SMA and collagen1-α* and ROS production and inhibited the expression level of P-Smad3C protein. Therefore, curcumin shows the potential to become a promising candidate for treating liver fibrosis.

## DECLARATIONS

### Acknowledgments

No artificial intelligence was used in this study.

### Ethical approval

The present study was designed and conducted with the permission of the Ethics Committee of Ahvaz Jundishapur University of Medical Sciences, Ahvaz, Iran (ethical code: IR.AJUMS.REC.1400.390).

### Consent to participate

Not applicable.

### Consent for publication

All authors reviewed the results and approved the final version of the manuscript.

### Authors’ contributions

SHA: designed the study; MH: performed all assays, contributed to the disease diagnosis and selection of patients and analyzed the data; SSB: analyzed the data; 

BK: performed all assays; ESH: wrote the first draft; MR: contributed to interpreting the results, designed the study and revised the manuscript.

### Data availability

 All relevant data can be found within the manuscript.

### Competing interests

The authors declare that they have no competing interests. 

### Funding

This project was financially supported by Ahvaz Jundishapur University of Medical Sciences, Ahvaz, Iran (grant number: CMRC-0038).

### Supplementary information

The online version does not contain supplementary material.
